# First Complete Genome Sequence of *Corynebacterium riegelii*

**DOI:** 10.1128/genomeA.00084-17

**Published:** 2017-03-30

**Authors:** Alexander L. Greninger, Jessica Streithorst, Charles Y. Chiu, Steve Miller

**Affiliations:** ^a^ Department of Laboratory Medicine, UCSF, San Francisco, California, USA; ^b^ Department of Laboratory Medicine, University of Washington, Seattle, Washington, USA

## Abstract

Here, we report the first complete genome sequence of *Corynebacterium riegelii* strain PUDD_83A45, isolated from the urine of a patient with urinary tract infection. The genome measured 2.56 Mb and contained no plasmid.

*Corynebacterium riegelii* is a newly described species of corynebacteria originally isolated in 1997 from pure cultures of urinary tract infections in females and is named after corynebacteriologist Philippe Riegel (1, 2). Since its discovery, it has been uniformly associated with urinary tract infections and twice from urosepsis (3, 4). Biochemically, it is distinguished by its strong urease positivity and ability to ferment maltose but not glucose (1). We sequenced the first complete genome of *C. riegelii* strain PUDD_83A45, isolated from a 2003 urine sample from a 9-year-old male with congenital hypospadias and hydronephrosis who presented with symptoms of urinary tract infection. The patient had multiple surgical repairs as a child to correct anatomical and obstructive urinary tract defects and had multiple urinary tract infections prior to this presentation. The isolate was identified biochemically and determined to be susceptible to penicillin and vancomycin by disk diffusion testing.

DNA from *C. riegelii* strain PUDD_83A45 was extracted using the Qiagen EZ1 kit. Nextera XT paired-end and Nextera mate-pair libraries were sequenced 2 × 300 bp and 2 × 80 bp, respectively, on an Illumina MiSeq platform. DNA was also sequenced using approximately half of a flow cell of an Oxford Nanopore Minion sequencer. Illumina sequences were adapter and quality (Q30) trimmed using Cutadapt or NxTrim, *de novo* assembled using SPAdes version 3.5, metagenomically screened for contaminating sequences with SURPI, and annotated via Prokka version 1.1 (5–10). A total of 2,265,156 paired-end reads and 4,092,796 mate-pair reads were recovered after trimming, while 1,381 “pass” and 26,418 “fail” reads were recovered from the Nanopore sequencer. *De novo* assembly combined with manual integration of Nanopore data via LASTZ alignments yielded a single scaffold of 2,563,736 bp, an average coverage of 225×, and a total of 2,395 coding sequences. Two gaps of <1.5 kb remained in the genome due to a transposase that was also present at 96% nucleotide identity in *Corynebacterium imitans* strain DSM 44264, as well as a highly repetitive sequence in a Rib surface antigen family protein.

BLASTn analysis of the complete 16S sequence from strain PUDD_83A45 showed 99.8% identity to *C. riegelii* strain CTF08-1967 (EU848548) and *C. riegelii* isolate 99-0185 (AF537602). BLASTn analysis of the complete *rpoB* gene demonstrated 98.8% identity to *C. riegelii* strain CIP 103310 (AY492278). The genome contained a 5.1-kb locus that included *ureA-G* genes that aligned 71 to 80% by nucleotide to known urealytic corynebacteria such as *C. pseudotuberculosis*, *C. ulcerans*, *C. urealyticum*, and *C. ureicelerivorans*. Annotated maltose-related genes included a *malF* maltose permease that aligned with 76% nucleotide identity to *C. ureicelerivorans* strain IMMIB RIV-2301 (CP009215) and a *malE* maltose-binding periplasmic protein that aligned with 76% nucleotide identity to *C. glutamicum* strain B253 (CP010451). No high-confidence antibiotic resistance genes were identified by Comprehensive Antibiotic Resistance Database analysis, although distant matches included two multidrug MFS transporters, one closest to *Actinomyces neuii* (63% amino acid identity to WP_024330734) and one closest to *Propionibacterium* sp. KPL1838 (73% amino acid identity to ERS38161) (11).

## Accession number(s).

This whole-genome shotgun project has been deposited at DDBJ/ENA/GenBank under the accession number CP012342.
